# Recent Insights Into the Structure, Function, and Evolution of the RNA-Splicing Endonucleases

**DOI:** 10.3389/fgene.2019.00103

**Published:** 2019-02-12

**Authors:** Akira Hirata

**Affiliations:** Department of Materials Science and Biotechnology, Graduate School of Science and Engineering, Ehime University, Matsuyama, Japan

**Keywords:** RNA-splicing endonuclease, intron-containing tRNA, broad substrate specificity, co-evolution of protein and RNA, archaea and eukaryote

## Abstract

RNA-splicing endonuclease (EndA) cleaves out introns from archaeal and eukaryotic precursor (pre)-tRNA and is essential for tRNA maturation. In archaeal EndA, the molecular mechanisms underlying complex assembly, substrate recognition, and catalysis have been well understood. Recently, certain studies have reported novel findings including the identification of new subunit types in archaeal EndA structures, providing insights into the mechanism underlying broad substrate specificity. Further, metagenomics analyses have enabled the acquisition of numerous DNA sequences of EndAs and intron-containing pre-tRNAs from various species, providing information regarding the co-evolution of substrate specificity of archaeal EndAs and tRNA genetic diversity, and the evolutionary pathway of archaeal and eukaryotic EndAs. Although the complex structure of the heterothermic form of eukaryotic EndAs is unknown, previous reports regarding their functions indicated that mutations in human EndA cause neurological disorders including pontocerebellar hypoplasia and progressive microcephaly, and yeast EndA significantly cleaves mitochondria-localized mRNA encoding cytochrome b mRNA processing 1 (Cpb1) for mRNA maturation. This mini-review summarizes the aforementioned results, discusses their implications, and offers my personal opinion regarding future directions for the analysis of the structure and function of EndAs.

## Introduction

Transfer RNAs (tRNAs) play a fundamental role as adapter molecules for mRNA translation. Maturation events in tRNAs, including removals of the 5′-leader, 3′-trailer, and intron sequences, modification, and addition of 3′-CCA sequences and amino acids are essential for protein synthesis. During tRNA maturation, tRNA splicing is one of the most significant processes in intron splicing and ligation of the two halves of exons in the precursor (pre)-tRNA. Pre-tRNA introns are either auto-catalytically or enzymatically cleaved out in the three domains of life. Group I introns found in pre-tRNA in some bacteria and higher eukaryotic plastids are auto-catalytically cleaved out with an external guanosine-5′-triphosphate (GTP) (Xu et al., 1990; Haugen et al., 2005). By contrast, the introns in cytoplasmic eukaryotic and archaeal pre-tRNAs are enzymatically cleaved out by an RNA-splicing endonuclease (EndA) (Abelson et al., 1998) and the two halves of the exon are subsequently ligated by a tRNA ligase (Phizicky et al., 1986; Westaway et al., 1988; Englert et al., 2011; Popow et al., 2011; Tanaka et al., 2011). Eukaryotic EndA has been extensively identified and characterized in yeast, xenopus, and human. The yeast and human isoform comprise four distinct subunits, referred to as either Sen2, Sen15, Sen34, and Sen54 or αβγσ (Rauhut et al., 1990; Trotta et al., 1997, 2006; Paushkin et al., 2004), although the complete structure of the heterothermic form of eukaryotic EndAs remains unknown. The intron cleavage mechanism of eukaryotic EndAs has been demonstrated owing to early advancements by Dr. John Abelson’s and Dr. Glauco Tocchini-Valentini’s groups (Reyes and Abelson, 1988; Baldi et al., 1992; Bufardeci et al., 1993). Furthermore, archaeal EndAs are classified into three types [α_4_, α′_2_, (αβ)_2_] in accordance with the subunit components (Tocchini-Valentini et al., 2005b) until the 𝜀_2_ type of archaeal End is newly identified and characterized (Fujishima et al., 2011; Hirata et al., 2012). Currently, four types of EndAs are found in archaea. The general mechanism underlying the recognition and cleavage of pre-tRNA by archaeal EndA was previously reported by Dr. John Abelson’s and Dr. Hong Li’s groups (Li et al., 1998; Li and Abelson, 2000; Xue et al., 2006). Eukaryotic EndA follows a similar mechanism, implicating an evolutionary association between archaeal and eukaryotic EndAs. Furthermore, Calvin and Li (2008) reported the molecular mechanisms underlying complex assembly, substrate recognition, and catalysis in archaeal EndA. Their review article still provides robust evidence regarding the mechanisms underlying substrate recognition and intron-cleavage by archaeal EndAs. This mini-review is focused on recent advancements regarding the structure, function, and evolution of archaeal and eukaryotic EndAs and additionally provides a perspective for future studies on the structure and function of EndAs.

## Structure

**FIGURE 1:**
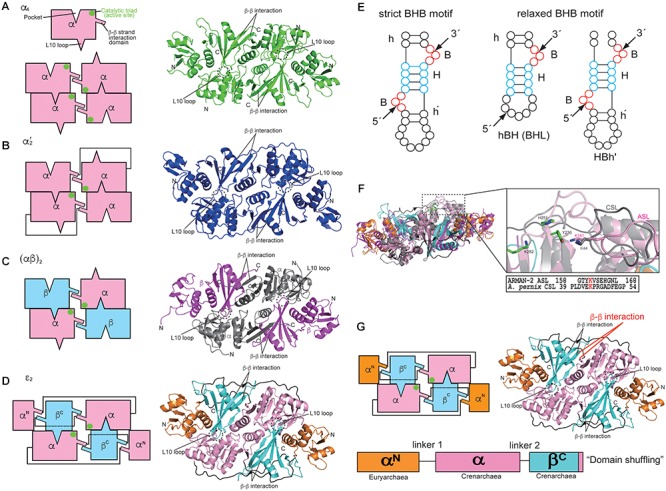
Structures and characteristics of four types of archaeal RNA-splicing endonucleases (EndAs): **(A)** α_4_ type *Methanocaldococcus jannaschii* EndA; **(B)** α′_2_ type *Archaeoglobus fulgidus* EndA; **(C)** (αβ)_2_ type *Aeropyrum pernix* EndA; **(D)** 𝜀_2_ type ARMAN-2 EndA. Interactions among the subunits are represented by cartoon models on the left side. The β–β interaction responsible for inter/intra-unit formation, the L10 loop and pocket responsible for dimer/tetramer formation are highlighted. The catalytic triads are marked by green circles. The right panels show the ribbon models of EndAs. **(E)** Left, strict BHB motif; Right, relaxed BHB motifs (hBH and HBh′). **(F)** Comparison of the ARMAN-2-specific loop (ASL) in the ARMAN-2 EndA and Crenarchaea-specific loop (CSL) in the *Aeropyrum pernix* EndA Left: superimposed structures of ARMAN-2 EndA and *Aeropyrum pernix* EndA. Ribbon diagram of the ARMAN-2 EndA and *Aeropyrum pernix* EndA are represented by colors similar to those in **(C,D)**. Right: close-up view of the structure of the ASL region (pink) of ARMAN-2 EndA superimposed on the structure of the CSL region (gray) of *Aeropyrum pernix* EndA. The catalytic triad comprised three catalytic residues (Y236, H251, and K282), shown by a stick model (green). Structure-based sequence alignment is shown at the bottom of the superimposed structures. The conserved K161 in ASL and K44 in CSL are highlighted in red. **(G)** Gene recombination of three units in the 𝜀 protomer of ARMAN-2 EndA. Interactions among units are represented by cartoon models on the left side. The panels on the right side show the ribbon models of EndAs. The β–β interactions responsible for inter/intra-unit formation are altered for gene recombination (red). These figures are illustrated with some modifications using previous figures (Hirata et al., 2012) and reproduced with permission based on the copyright policy from Oxford University Press.

Information regarding the four types of archaeal EndA structures, i.e., α_4_, α′_2_, (αβ)_2_, and 𝜀_2_, has been obtained from extensive crystallographic studies (Table 1), whereas only the structure of one subunit (Sen15) of eukaryotic EndA has been determined by nuclear magnetic resonance (NMR) spectroscopy (Song and Markley, 2007). Initially, Dr. John Abelson’s group determined the X-ray structure of the homotetrameric form (α_4_) of archaeal EndA in *Methanocaldococcus jannaschii* (Li et al., 1998) and of the homodimeric form (α′_2_) in *Archaeoglobus fulgidus* (Li and Abelson, 2000). The α′_2_ type of EndA has also been determined in *Thermoplasma acidophilum* by another group (Kim et al., 2007). The overall structures of two types are suggestive of a rectangular parallelepiped conformation (Figure 1A,B). Briefly, the N-terminal domain of one α subunit in α_4_ type of archaeal EndA consists of three α helices and a mixed antiparallel/parallel β sheet, and the C-terminal domain comprises two α helices and a central four-stranded mixed β sheet. Homotetramer formation is achieved by two significant interactions: interaction between two β–β strands at the domain interface between two α subunits and interaction between a negatively charged L10 loop of the α subunit with a positively charged pocket of the opposing α subunit. The interactions are conserved in the four types of archaeal EndAs. The α subunit of α′_2_ type of EndA is considered the fusion protein of two α subunits of α_4_ type of EndA because of the evolutionary association between the α_4_ and α′_2_ types, based on their sequence similarity, and the two α subunits are connected by a linker from the C-terminal domain of α subunit to the N-terminal domain of another subunit. X-ray structures of (αβ)_2_ type of archaeal EndAs have been reported in *Nanoarchaeum equitans* (Mitchell et al., 2009), *Pyrobaculum aerophilum* (Yoshinari et al., 2009), *Aeropyrum pernix* (Hirata et al., 2011; Okuda et al., 2011), and *Methanopyrus kandleri* (Kaneta et al., 2018). The (αβ)_2_ type EndA comprises two α catalytic subunits and two β structural subunits, and the four subunits are assembled into a heterotetramer (αβ)_2_ through the aforementioned interactions. The overall structures are very similar to those of the α_4_ and α′_2_ types of EndAs, although the structure of *P. aerophilum* EndA is more compact than that of other EndAs because of the absence of the N-terminal domain of structural β subunit. Furthermore, a new type of 𝜀_2_ EndA was identified and characterized in *Candidatus Micrarchaeum acidiphilum* (ARMAN-2) (Fujishima et al., 2011; Hirata et al., 2012), which is deeply branched within Euryarchaeota. ARMAN-2 EndA forms an 𝜀_2_ homodimer through evolutionarily conserved interactions in the other three types of archaeal EndAs. The 𝜀 protomer is very unique and is separated into three units (α^N^, α, and β^C^) fused by two distinct linkers, although the overall shape of ARMAN-2 𝜀_2_ EndA is similar to that of the other three types of archaeal EndAs. Structure-based sequence analysis suggests that all four types of archaeal EndAs evolved from a common ancestor.

**Table 1:** Structural and functional characterization of archaeal and eukaryotic EndAs.

Species	Functional subunits	Amino acid length (aa)	Specificity	PDB_ID	Reference
*Methanocaldococcus jannaschii*	α_4_	α = 179	Narrow	1A79	Li et al., 1998
*Archaeoglobus fulgidus*	α′_2_	α = 305	Narrow	1RLV 2GJW (RNA complex)	Li and Abelson, 2000; Xue et al., 2006
*Thermoplasma acidophilum*	α′_2_	α = 289	Narrow	2OHC	Kim et al., 2007
*Aeropyrum pernix*	(αβ)_2_	α = 186, β = 170	Broad	3P1Z 3AJV (H133A)	Hirata et al., 2011; Okuda et al., 2011
*Nanoarchaeum equitans*	(αβ)_2_	α = 154, β = 153	Broad	3IEY	Mitchell et al., 2009
*Pyrobaculum aerophilum*	(αβ)_2_	α = 183, β = 96	Broad	2ZYZ	Yoshinari et al., 2009
*Methanopyrus kandleri*	(αβ)_2_	α = 179, β = 166	Constrained broad	5X89	Kaneta et al., 2018
*Candidatus Micrarchaeum acidiphilum* (ARMAN-2)	𝜀_2_	𝜀 = 390	Broad	4FZ2	Hirata et al., 2012
					
*Saccharomyces cerevisiae*	α (Sen2), β (Sen34), γ (Sen15), δ (Sen54)	α = 377, β = 275 γ = 128, δ = 467	Broad?		Trotta et al., 1997
*Homo sapience*	α (TSen2), β (TSen34), γ (TSen15), δ (TSen54)	α = 465, β = 310 γ = 171, δ = 526	Broad?	2GW6 (TSen15)	Song and Markley, 2007


Three catalytic residues (tyrosine, histidine, and lysine) are conserved in the four types of EndAs, and each subunit assembly of the archaeal EndAs leads to the formation of two intron cleavage sites at the active site (Figure 1A–D, green circle). Similarly, two sets of the two substrate recognition residues [two arginines in α subunit of α4 and α′_2_ types or arginine and tryptophan residues in α subunit of (αβ)_2_ and 𝜀_2_ types] are positioned at a similar location adjacent to the three catalytic residues. Thus, each multimeric conformation of archaeal EndAs is essential for catalysis and substrate tRNA recognition. In eukaryotes, yeast EndA is a heterotetramer (αβγσ) comprising two catalytic (Sen2 and Sen34) and two accessory (Sen15 and Sen54) subunits identified on the basis of homology with their human counterparts (Trotta et al., 1997). The Sen2 and Sen34 share homology with the α subunit of archaeal EndAs and employ the catalytic residues (histidine, tyrosine, and lysine) identical to their archaeal counterparts. Therefore, eukaryotic and archaeal EndAs are presumed to employ a molecular mechanism of cleavage similar to that of ribonuclease A, using the three catalytic residues (Raines, 1998; Calvin and Li, 2008). The complex structure of the heterodimer in eukaryotic EndA is unknown, although the NMR structure of human Sen15 is known (Table 1). The structural arrangement of human Sen15 is similar to that of the C-terminal domain of the α subunit in *M. jannaschii* α_4_ EndA. Together, these findings implicate an evolutionary relationship between the eukaryotic and archaeal isoforms of EndA.

## Substrate Specificity

Initial studies on the substrate specificity of archaeal EndAs were conducted by Dr. Charles Daniels’ and Roger Garrett’s groups (Kjems and Garrett, 1988; Thompson and Daniels, 1988; Palmer et al., 1992; Kleman-Leyer et al., 1997; Lykke-Andersen and Garrett, 1997; Lykke-Andersen et al., 1997a,b). Archaeal EndAs are known to recognize a bulge-helix-bulge (BHB) motif (Figure 1E), which comprises two bulges (3 nt) separated by one helix (4 nt) located at the exon–intron boundary of pre-tRNAs (Marck and Grosjean, 2003). The canonical BHB motif is frequently present in the anticodon loop between position 37 and 38 (37/38) of archaeal pre-tRNA; however, in some cases, this motif is present in pre-mRNA and pre-rRNA for their maturation (Kjems and Garrett, 1991; Yoshinari et al., 2006). In contrast with this canonical BHB motif, two types of relaxed BHB motifs, non-canonical introns (hBH and HBh′), are present in pre-tRNAs (Figure 1E). The relaxed BHB motifs of hBH and HBh′ disrupt either 5′ or 3′ bulges in the canonical BHB motif. One of the bulges is often absent to form a relaxed bulge-helix-loop (BHL). Furthermore, the unique features of disrupted tRNA genes include multiple (two or three) intron-containing tRNAs (Sugahara et al., 2008; Tocchini-Valentini et al., 2009), split and tri-split tRNAs, wherein tRNA fragments are encoded by two or three genes (Randau et al., 2005; Fujishima et al., 2009), and permuted tRNAs, wherein the sequences of 5′ and 3′ halves of tRNA genes are inverted (Chan et al., 2011). Remarkably, the canonical and relaxed BHB motifs are located not only at the anticodon loop position 37/38 but also at the D-loop, T-loop, and acceptor-stem of archaeal pre-tRNAs (Marck and Grosjean, 2003; Yoshihisa, 2014). Although introns with canonical and relaxed BHB motifs are distributed at the various positions in pre-tRNA, archaeal EndAs actually recognize and cleave introns. However, only two types, i.e., (αβ)_2_ and 𝜀_2_ EndAs, can efficiently eliminate introns with relaxed BHB motifs, thereby displaying broad substrate specificity in the EndAs. Eukaryotic EndA recognizes and eliminates introns with a canonical BHB motif from archaeal pre-tRNA, although in most eukaryotic pre-tRNAs, the introns are located at the anticodon loop 37/38 and includes the BHL motif. To eliminate introns with a BHL motif, eukaryotic EndA requires a mature domain of pre-tRNA, wherein the interaction between the D- and T-loops yields a unique structure, the so-called “elbow” (Reyes and Abelson, 1988; Calvin and Li, 2008). The α′_2_ type of archaeal EndA from *Archaeoglobus fulgidus* can eliminate introns with the BHL motif at position 37/38 in the case of full-length pre-tRNA (Tocchini-Valentini et al., 2005a).

## Broad Substrate Specificity of the Archaeal EndAs

The (αβ)_2_ and 𝜀_2_ EndAs have broad substrate specificity, which can efficiently cleave not only the introns with canonical BHB motif but also those with a relaxed BHB motif. The molecular mechanism underlying the broad substrate specificity of (αβ)_2_ EndA is unknown. To clarify the mechanism, structural and biochemical analyses of the (αβ)_2_ type of EndA from hyperthermophilic crenarchaeon *Aeropyrum pernix* was performed (Hirata et al., 2011). At the time, (αβ)_2_-type EndAs were reported exclusively in crenarchaea and nanoarchaea, except for euryarchaeon *Methanopyrus kandleri* (Marck and Grosjean, 2003). Our studies on *A. pernix* EndA reported a Crenarchaea-specific loop (CSL), which was conserved in crenarchaeal EndAs and located adjacent to the active site (Figure 1F). Furthermore, insertion of CSL in *A. fulgidus* α′_2_ EndA conferred *A. pernix* EndA with broad substrate specificity, which originally had narrow substrate specificity. In the *A. pernix* EndA with a CSL insert, an alanine-substituted mutant of the conserved Lys residue of CSL disrupted the broad substrate specificity. Together, these findings suggest that the Lys residue of CSL plays a significant role as an RNA binding site and is responsible for the broad substrate specificity in the (αβ)_2_ of crenarchaeal EndAs. Similarly, the 𝜀_2_ type of ARMAN-2 EndA possesses an ARMAN-2 specific loop (ASL), which confers broad substrate specificity, and the Lys residue of ASL functions as the RNA recognition site. Although the ASL conformation in ARMAN-2 EndA is markedly similar to that of CSL in *A. pernix* EndA, there are no obvious sequence similarities between the ASL and CSL, except for the conserved Lys residue, which functions as the substrate recognition site. Together, these findings indicate that the ASL was acquired by a distinctly independent evolutionary pathway toward the CSL (i.e., “convergent evolution”). However, it is still unknown why each Lys residue conserved in the CSL and ASL is required for intron cleavage, despite the presence of three catalytic residues in the EndAs. However, *M. kandleri* EndA was identified as the (αβ)_2_ type lacking specific loops such as the ASL and CSL (Kaneta et al., 2018). While *M. kandleri* EndA slightly cleaves introns with a relaxed BHB motif in *M. kandleri* pre-tRNA^Glu^ (UUC), it could not eliminate introns from a mini-helix RNA with a BHL motif. Therefore, the *M. kandleri* EndA is considered to be of the (αβ)_2_ type with constrained substrate specificity.

## Evolution

The α_4_ type of archaeal EndA, which encodes a single catalytic α subunit, is proposed to be the prototype of the EndAs (Tocchini-Valentini et al., 2005b), and the subsequent subfunctionalization of gene duplication and fusion has yielded the other three types [α′_2,_ (αβ)_2_ and 𝜀_2_]. Intriguingly, 𝜀_2_-type ARMAN-2 EndA appears to have undergone a genetic recombination of the three subunits, euryarchaeal α subunit, crenarchaeal α subunit, and crenarchaeal β subunit (Hirata et al., 2012), comprising three units (α^N^-α-β^C^) of the 𝜀 protomer (Figure 1G). Each unit is clearly divided into a domain structure, thus providing a good example of the so-called “domain shuffling” occurring naturally. Moreover, the C-terminal subdomain of the crenarchaeal β subunit may have been incorporated into the terminus of the crenarchaeal α subunit, which may have primarily led to changes in the structural location of β–β interaction responsible for subunit assembly.

The sequence of archaeal α subunit is locally conserved in the two catalytic subunits (Sen2 and Sen34) of the heterotetrameric form (αβγδ) of eukaryotic EndA with approximately 50 amino acid residues. Therefore, eukaryotic EndA is considered to have evolved from the archaeal (αβ)_2_ EndA with the acquisition of new subunits (γ and δ). Remarkably, the primitive eukaryotic red alga *Cyanidioschyzon merolae* harbors many disrupted tRNA genes with a relaxed BHB motif as employed in Archaea (Soma et al., 2007, 2013; Soma, 2014). The *C. merolae* EndA is expected to comprise three subunits [cmSen2p, cmSen34p, and cmSen54p (αβγ)] for processing these pre-tRNAs; however, it does not contain the ASL and CSL. Thus, heterotrimer form of *C. merolae* EndA might be an intermediate in the evolutionary transition between the heterotetramer of archaeal EndA to heterotetramer of eukaryotic EndA. Furthermore, recent bioinformatics analysis has reported that archaeal species with specific loops such as the ASL and CSL in EndAs clearly represent a trend of increased intron-containing tRNA genes with BHB and relaxed BHB motifs, suggesting coevolution of tRNA gene diversity and broad substrate specificity (Kaneta et al., 2018). These findings further update the previous concept of co-evolution (Tocchini-Valentini et al., 2005b; Fujishima and Kanai, 2014).

## New Aspects of Eukaryotic EndA

Vertebrate and *Saccharomyces cerevisiae* EndAs are localized in the nucleus (Paushkin et al., 2004) and on the mitochondrial outer membrane (Yoshihisa et al., 2003, 2007), respectively. A recent study reported that S. *cerevisiae* EndA cleaves the mitochondria-localized mRNA encoding Cbp1 (cytochrome *b* mrNA processing 1) and this cleavage requires a predicted stem-loop structure of the endonucleolytic cleavage-inducible sequence of Cpb1 with synergistic effects of other factors (Tsuboi et al., 2015). These significant findings provide evidence regarding the biological role of mitochondrial-localized S. *cerevisiae* EndA and suggest that the EndA has broad substrate specificity owing to specific recognition of the predicted stem-loop structure without the BHB motif. Furthermore, the human EndA complex (TSen2, TSen15, TSen34, and TSen54) reportedly cleaves introns from pre-tRNAs, and the TSen2 subunit is involved in pre-mRNA′3 end formation (Paushkin et al., 2004). These reports further expand the possibility that the substrates of EndA are non-coding RNAs involved in the regulation of gene expression. To confirm the possibility, crosslinking RNA-EndA complex using UV irradiation combined with immunoprecipitation and RNA sequencing could be a useful method to identify the non-coding RNAs as the substrate of EndA. More importantly, recessive mutations in the genes of three subunits (TSen2, TSen34, and TSen54) cause pontocerebellar hypoplasia (PCH) types 2A-C, 4, and 5 (Budde et al., 2008; Namavar et al., 2011a,b; Bierhals et al., 2013; Maraş-Genç et al., 2015). PCH2 is reportedly involved in progressive cerebral atrophy and microcephaly, dyskinesia, seizures and early childhood mortality. Furthermore, a recent study reported that three homozygous *TSEN15* cause a milder version of the PCH2-related pathology (Breuss et al., 2016). Hence, appropriate EndA function is required for brain development in humans. However, the mechanism underlying the pathogenesis of PCH which is caused by human EndA mutations remains unclear because its complex structure is yet unknown.

## Conclusion and Future Perspectives

The mechanism underlying the recognition and cleavage of RNA introns by EndAs is known; however, they have gained increasing interest, since the evolutionary pathway from archaeal to eukaryotic EndA and the mechanism underlying the broad substrate specificity of archaeal and eukaryotic EndA warrant further investigation. The conserved Lys residue in CSL and ASL of the (αβ)_2_ and 𝜀_2_ types of archaeal EndAs might function as the catalytic and RNA recognition residue. Eukaryotic EndAs probably possess broad substrate specificity, similar to the archaeal (αβ)_2_- and 𝜀_2_- type EndAs, whereas the mechanism underlying the broad substrate specificity may vary between the eukaryotic and archaeal EndAs. Further structural analysis is required to elucidate the detailed mechanism underlying broad substrate specificity by archaeal and eukaryotic EndAs. In particular, the structural information of human EndA may be useful for drug design that improves the inadequate EndA function, which causes the developmental retardation of human brain described above.

## Author Contributions

The author confirms being the sole contributor of this work and has approved it for publication.

## Conflict of Interest Statement

The author declares that the research was conducted in the absence of any commercial or financial relationships that could be construed as a potential conflict of interest.
